# NOD1 deficiency ameliorates the progression of diabetic retinopathy by modulating bone marrow–retina crosstalk

**DOI:** 10.1186/s13287-024-03654-y

**Published:** 2024-02-09

**Authors:** Jingwen Qiu, Jing Wu, Wenwen Chen, Yu Ruan, Jingning Mao, Shue Li, Xuan Tang, Lei Zhao, Shengbing Li, Ke Li, Dongfang Liu, Yaqian Duan

**Affiliations:** 1 Department of Endocrinology and Metabolism, The Second Affiliated Hospital of Chongqing Medical University, Chongqing, China; 2 Department of Hematology/Oncology, Chongqing University Cancer Hospital, Chongqing, China; 3 Division of Growth, Development and Mental Health of Children and Adolescence, Children’s Hospital of Chongqing Medical University, Chongqing, China; 4 Health Medical Center, The Second Affiliated Hospital of Chongqing Medical University, Chongqing, China; 5 Department of Laboratory Medicine, The Second Affiliated Hospital of Chongqing Medical University, Chongqing, China; 6 Center for Lipid Research, Key Laboratory of Molecular Biology for Infectious Diseases, Ministry of Education, The Second Affiliated Hospital of Chongqing Medical University, Chongqing, China

**Keywords:** Nucleotide-binding oligomerization domain-containing protein 1, Diabetic retinopathy, Hematopoietic stem/progenitor cells, Macrophage, Inflammation

## Abstract

**Background:**

Nucleotide-binding oligomerization domain-containing protein 1 (NOD1) plays a pivotal role in inducing metabolic inflammation in diabetes. Additionally, the NOD1 ligand disrupts the equilibrium of bone marrow-derived hematopoietic stem/progenitor cells, a process that has immense significance in the development of diabetic retinopathy (DR). We hypothesized that NOD1 depletion impedes the advancement of DR by resolving bone marrow dysfunction.

**Methods:**

We generated NOD1^−/−^-Akita double-mutant mice and chimeric mice with hematopoietic-specific NOD1 depletion to study the role of NOD1 in the bone marrow–retina axis.

**Results:**

Elevated circulating NOD1 activators were observed in Akita mice after 6 months of diabetes. NOD1 depletion partially restored diabetes-induced structural changes and retinal electrical responses in NOD1^−/−^-Akita mice. Loss of NOD1 significantly ameliorated the progression of diabetic retinal vascular degeneration, as determined by acellular capillary quantification. The preventive effect of NOD1 depletion on DR is linked to bone marrow phenotype alterations, including a restored HSC pool and a shift in hematopoiesis toward myelopoiesis. We also generated chimeric mice with hematopoietic-specific NOD1 ablation, and the results further indicated that NOD1 had a protective effect against DR. Mechanistically, loss of hematopoietic NOD1 resulted in reduced bone marrow-derived macrophage infiltration and decreased CXCL1 and CXCL2 secretion within the retina, subsequently leading to diminished neutrophil chemoattraction and NETosis.

**Conclusions:**

The results of our study unveil, for the first time, the critical role of NOD1 as a trigger for a hematopoietic imbalance toward myelopoiesis and local retinal inflammation, culminating in DR progression. Targeting NOD1 in bone marrow may be a potential strategy for the prevention and treatment of DR.

**Supplementary Information:**

The online version contains supplementary material available at 10.1186/s13287-024-03654-y.

## Background

Diabetic retinopathy (DR) is a prominent microvascular complication of diabetes and ranks as the primary cause of blindness among working-age adults in developed nations [1]. Present treatments include intravitreal anti-VEGF injections, laser photocoagulation, and vitrectomy [2]. These therapeutic choices primarily address advanced stages of DR once vision is compromised, potentially lacking universal efficacy and offering limited options during the early phases of the condition. Consequently, it has become imperative to delve deeper into the cellular and molecular mechanisms underpinning the pathobiology of DR progression.

Accumulating evidence suggests that diabetes compromises the integrity of the intestinal barrier, facilitating the translocation of microorganisms, their metabolites, and pathogen-associated molecular patterns (PAMPs), including lipopolysaccharide (LPS) and peptidoglycan (PGN), into the bloodstream [3]. PAMPs and their receptors have garnered attention with regard to diabetes pathogenesis due to their capacity to incite an immune response and provoke chronic low-grade inflammation upon entry into circulation, potentially culminating in insulin resistance [4]. Numerous studies underscore the association between elevated circulating LPS, a well-studied PAMP, and the advancement of diabetic microvascular complications [5]. Both chronic and acute administration of LPS in animal models mimics the pathological hallmarks of diabetic retinopathy (DR) or exacerbates existing retinopathy in diabetes by targeting Toll-like receptor 4 (TLR4) [6, 7]. Interestingly, our recent investigations have identified the accumulation of another type of PAMP, PGN, in the plasma of both type 1 and type 2 diabetic patients as well as in diabetic mouse models [8, 9]. Furthermore, nucleotide-binding oligomerization domain-containing protein 1 (NOD1), a widely expressed intracellular receptor for PGN, has been recognized as a pivotal sensor in triggering metabolic inflammation and insulin resistance in diabetes [10]. However, the precise role of NOD1 in the pathogenesis of diabetic microvascular complications, such as DR, remains largely unexplored.

The disruption of bone marrow hematopoiesis, leading to a reduction in circulating angiogenic cells with vessel reparative potential and an increase in proinflammatory cell types, constitutes a pivotal factor in the evolution of DR [11, 12]. Intriguingly, results from our prior research demonstrate that the NOD1 ligand detrimentally impacts both the quantity and functionality of stem and progenitor cell populations within the diabetic bone marrow [9]. Additionally, the results of other studies have suggested that NOD1 activation is involved in regulating hematopoietic stem cell (HSC) self-renewal and steering differentiation toward myeloid lineages [10, 13, 14]. We posit that recognition by NOD1 of pathogen-associated molecular patterns derived from bacteria prompts an imbalance in hematopoiesis and an augmented influx of proinflammatory cells derived from the bone marrow into the retina. Consequently, this sequence ultimately contributes to the progression of DR.

## Methods

### Animals

Ethics approval for all animal procedures was obtained by the Animal Research Committee at Chongqing Medical University, and all procedures were conducted in accordance with established standard protocols for animal care. The animal studies presented in this manuscript were reported in compliance with the ARRIVE guidelines. In general, distinct genotypes of mice were utilized for this study: C57BL/6J wild type (WT, genotype: *NOD1*^+/+^:*Ins2*^+/+^), Akita (genotype: *NOD1*^+/+^:*Ins2*^+/Akita^), NOD1 knockout (NOD1^−/−^, genotype: *NOD1*^−/−^:*Ins2*^+/+^), and NOD1 knockout Akita double mutant (NOD1^−/−^-Akita, genotype: *NOD1*^−/−^:*Ins2*^+/Akita^). C57BL/6J WT and homozygous *NOD1*^−/−^ mice were purchased from Cyagen Biosciences. Male heterozygous diabetic Akita mice were obtained from Shanghai Model Organisms Center. The mice were carefully bred and nurtured at the animal facilities of both Cyagen Biosciences and Chongqing Medical University. Male Akita mice were crossed with female NOD1^−/−^ mice to generate the F1 generation, which was then bred with NOD1^−/−^ mice to obtain NOD1^−/−^-Akita double mutants. Given that male Akita mice manifest a markedly more severe diabetic phenotype than do female mice [15], male mice were exclusively employed for this study. We used resource equation to calculate the sample size of mice. Based on the “3R” principle of animal experiments, the number of each group was finally determined.

Body weights and random blood glucose levels were recorded every 2 weeks. Diabetic mice were examined at the 6-month mark after diabetes onset. Glycated hemoglobin was quantified using the A1CNow + kit (Bayer HealthCare, Sunnyvale, CA, USA) at the conclusion of the study. Upon completion of the study, mice were anesthetized and humanely euthanized through inhalation of an overdose of isoflurane, followed by cervical dislocation.

### Cell-based NOD1 activity reporter assay

HEK-Blue-mNOD1 HEK293 cells containing the murine NOD1 gene along with a reporter gene for secreted embryonic alkaline phosphatase were procured from InvivoGen (cat#: hkb-mnod1, San Diego, CA, USA). These cells were seeded and cocultured with HEK-Blue detection medium (cat#: hb-det2, InvivoGen, San Diego, CA, USA) alongside murine or human serum samples within a 96-well plate. Following 24 h of incubation, NOD1 stimulation was assessed utilizing a spectrophotometer at 630 nm.

### Optical coherence tomography (OCT)

OCT scans were performed using the InVivoVue imaging system (Bioptigen, Inc., NC, USA) to monitor retinal morphological changes in vivo. Mice were anesthetized by intraperitoneal injection of pentobarbital sodium (40 mg/kg). After dilation of the eyes with a 1% tropicamide solution, multiple high-resolution horizontal and vertical images were acquired per lesion, as detailed previously [14]. OCT imaging was performed in high-definition circular scanning mode. Total retinal thickness was defined as the distance from the distal edge of the retinal pigment epithelium (RPE) layer to the proximal edge of the nerve fiber layer (NFL). The retinal layers were further divided into the inner retinal layer (IRL) (from the NFL to the outer edge of the inner core layer (INL)) and the outer retinal layer (ORL) (from the INL terminal to the RPE). Retinal thickness measurements were taken at various distances from the optic disk (OD)—specifically at intervals of 150, 300, 600, and 900 µm in dorsal–ventral and nasal–temporal sectors as previously reported [16].

### Electroretinography (ERG)

The electrical responses of mouse retina were measured by ERG using a UTAS- E 2000 ERG system (LKC Technologies, MD, USA) as previously described with minor modifications [15]. After adaptation to the dark overnight, the mice were anesthetized with pentobarbital sodium (40 mg/kg), the pupils were dilated with 2.5% phenylephrine hydrochloride and 1% atropine sulfate ophthalmic solution, and the mice were placed on a temperature-regulated heating pad during recording periods. The recording ring electrode was placed in the center of the corneal surface, the grounding electrode was attached to the tail root, and the negative electrode was placed in the mouth of each mouse. The scotopic responses were assessed through 10 ascending stimulus intensities of light (0.0001, 0.001, 0.01, 0.1, 1, 3 cds/m^2^). After 3 min of adaptation to brightness, photopic responses were assessed utilizing 3 increasing light intensities (0.1, 1, 3 cds/m^2^). The light intensity was the average of three flashes 30 s apart. The next flash stimulus was used for OP_S_. The A-wave amplitude was measured from the first lowest peak to the prestimulus baseline after the flash began, and the B-wave amplitude was measured between the first lowest peak and the first peak. The oscillating potentials (OPs) were automatically filtered by ERG software. The latency and amplitude of OP1-3 were measured. For the flicker ERG, standard flash luminance with frequency (12 Hz) was obtained without any background illumination. The amplitude of flicker ERG is described as the lowest point of the waveform to the highest point.

### Acellular capillary quantification

The retinal vasculature was prepared through trypsin digestion, as previously described [17]. Briefly, retinas were fixed overnight using 4% paraformaldehyde, followed by incubation in 3% trypsin 250 (BD Biosciences, San Jose, CA, USA) on a gentle shaker at 37 °C. After each 30-min trypsin incubation interval, the retinas were rinsed with PBS until the internal limiting membrane was digested, leaving only the retinal vasculature. Subsequently, the retinal vasculature was meticulously mounted onto a glass slide and stained using periodic acid-Schiff’s base (PAS)-hematoxylin (Sigma-Aldrich, MO, USA). A Leica DM300 microscope was employed to capture retinal images, with acellular capillaries quantified across 10 randomly selected fields by two independent blinded investigators.

### Hematoxylin–eosin (HE) Staining

The right eyeball of mice was extracted and fixed in FAS eyeball solution (Servicebio, G1109) at room temperature for 24 h. Following fixation, the tissue was dehydrated using graded ethanol solutions and subsequently embedded in paraffin wax. Horizontal sections, 4 μm thick, were obtained from the optic nerve head using a microtome. These sections were then stained using standard H&E staining for morphological examination. The stratification of the entire retina, including the inner and outer layers, aligned with that observed in OCT imaging. Thickness measurements were taken using Image-pro Plus software.

### Bone marrow transplantation

Chimeric mice were established by bone marrow transplantation with C57BL/6J WT and Akita mice, following a previously outlined method with slight adaptations [10, 18]. Briefly, 8-week-old male recipient mice were lethally irradiated at a total dose of 11 Gy, after which they were transplanted with 5 × 10^6^ bone marrow cells from either NOD1^+/+^ or NOD1^−/−^ donors of the same age. Four distinct groups were established: WT → WT, NOD1^−/−^ → WT, Akita → Akita, and NOD1^−/−^-Akita → Akita. These groups were maintained until the study endpoint, which was set at 6 months after diabetes onset.

### Flow cytometry

Bone marrow cells were collected from mouse femurs and lysed with ammonium chloride solution (ACS). Retinal samples from six mice within the same group were pooled and digested with collagenase D (2 mg/mL) in a 37 °C incubator for 45 min, adhering to established protocols [19]. Isolated cells were then stained with primary antibody cocktails.

For bone marrow HSPC populations, the following antibodies were used as previously described [8]: anti-mouse CD127 (BD Biosciences, Cat# 562419); anti-mouse lineage antibodies (Biolegend, Cat# 133311); anti-mouse Sca-1 (Biolegend, Cat# 108114); anti-mouse c-Kit (Biolegend, San Diego, CA, Cat# 105806); anti-mouse CD135 (eBioscience, San Diego, CA, Cat# 46-1351-82); anti-mouse CD34 (Biolegend, Cat# 119308) and anti-mouse CD16/CD32 (eBioscience, Cat# 14–0161–82). For bone marrow immune cells and monocytes, the following primary antibodies were used: anti-mouse CD45 (BD Biosciences, Cat# 560510); anti-mouse NK 1.1 (BD Biosciences, Cat# 551114); anti-mouse CD3 (BD Biosciences, Cat# 560771); anti-mouse CD115 (Biolegend, Cat# 135513); anti-mouse Ly6G (BD Biosciences, Cat# 551461); anti-mouse Ly6C (Biolegend, Cat# 128006); and anti-mouse CD11b (Invitrogen, Cat# RM2817).

For retinal cells, the antibody cocktails comprised anti-mouse CD45 (BD Biosciences, Cat# 560510), anti-mouse CD11b (Invitrogen, Cat# RM2817), anti-mouse Ly6G (BD Biosciences, Cat# 551461), anti-mouse Ly6C (Biolegend, Cat# 128006), and F4/80 (Biolegend, Cat# 123116). The samples were stained with the primary antibody cocktails at 4 °C for 30 min in the dark, followed by staining with Flexible Viability Dye for an additional 30 min at 4 °C. Cells fixed with 1% PFA were subsequently analyzed using the LSR II flow cytometer and assessed using FlowJo software (V10).

### Colony-forming unit assay

ACS-lysed bone marrow cells were plated in MethoCult™ GF M3434 (STEMCELL Technologies) in accordance with the manufacturer’s instructions. A 1.1 ml MethoCult mixture was dispensed into a 35-mm petri dish. The final concentration of bone marrow cells in each dish for every group was 5 × 10^3^.

### Quantitative RT‒PCR

Retinal RNA was extracted using the RNeasy Mini Kit (Qiagen, Valencia, CA) and quantified using a Nanodrop (Thermo Scientific, Wilmington, DE). Subsequently, cDNA was synthesized with the iScript™ cDNA synthesis kit (Bio-Rad, Pleasanton, CA) following the manufacturer’s instructions. Quantitative PCR was conducted using SYBR Green with predesigned primers (Qiagen, cat#: 249900) on a StepOne Plus Real-Time PCR System (Life Technologies).

### Isolation and culture of bone marrow-derived macrophages (BMDMs)

Mouse femur bone marrow was flushed using PBS. The extracted bone marrow cells were cultured and differentiated into BMDMs as previously described [20]. BMDMs from WT → WT, NOD1^−/−^ → WT, Akita → Akita, and NOD1^−/−^-Akita → Akita chimeras were treated with either 50 μg/mL NOD1 ligand, PGN (Cat#: 77140, Sigma‒Aldrich, St Louis, MO), or an equivalent volume of vehicle for 24 h.

### ELISA

The expression of neutrophil elastase (NE) and myeloperoxidase (MPO) in the retina was measured using ELISA kits from R&D Systems. Pooled retina samples (5 retinas per sample) were prepared at a 1:1000 dilution for analysis. CXCL1 and CXCL2 secretion in BMDM culture supernatant was quantified using R&D Systems ELISA kits. Absorbance readings for test samples and standards were recorded by a microplate reader at 450 nm and subsequently adjusted with readings at 540 nm, following the manufacturer's instructions.

### Human blood samples

This study was approved by the Ethics Committee of the Second Affiliated Hospital of Chongqing Medical University. Sample collection was conducted in strict accordance with the tenets of the Declaration of Helsinki and the ARVO statement on human subjects. Peripheral blood was collected from age- and sex-matched groups: healthy controls (HC, *n* = 12), individuals with diabetes without microvascular complications (DM-NC, *n* = 8), and individuals with diabetes with nonproliferative diabetic retinopathy (NPDR, *n* = 8). The diagnosis of DR was established by proficient ophthalmologists based on fundus images.

### Data analysis and statistics

All results were statistically analyzed by GraphPad Prism software (version 8.3.0). Student’s t test was utilized for pairwise comparisons, and one-way or two-way ANOVA was employed for multiple comparisons, followed by appropriate post hoc tests. For nonnormally distributed data, the nonparametric Mann‒Whitney or Kruskal‒Wallis tests were utilized for statistical analysis. P values less than 0.05 (*p* < 0.05) were considered statistically significant. All data are presented as the mean ± standard deviation (SD).

## Results

### Elevated levels of NOD1 activators were observed in Akita mice

**Fig. 1:**
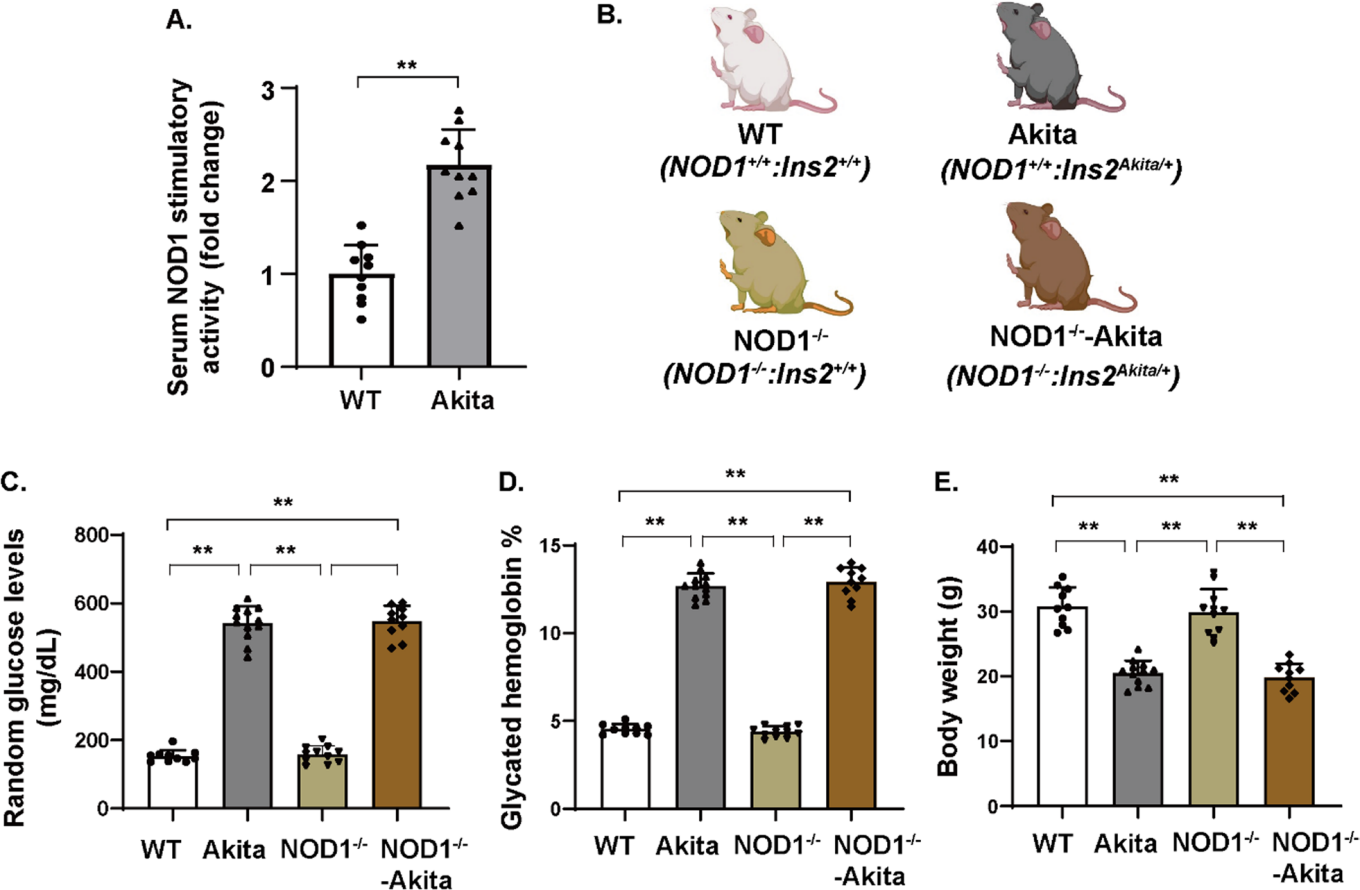
Increased circulating NOD1 activators in Akita mice. **A** NOD1 stimulatory activity was assessed in serum from Akita mice (6 months after diabetes onset) and age-matched controls using a HEK293T cell-based reporter assay (*n* = 10 per group). **p < 0.01. **B** Scheme for mouse strains. **C**–**E** Glycated hemoglobin, random blood glucose, and body weight were measured for Akita mice, NOD1^−/−^-Akita mice, and mice in the nondiabetic groups (*n* = 10–12 per group). The results are presented as the mean ± SD; ^#^*p* < 0.05 compared with WT; ^&^*p* < 0.05 compared with NOD1^−/−^. WT, wild type

Previous findings indicated that there was an increase in a NOD1 ligand, PGN, in both type 1 and type 2 diabetic mouse models [8, 9]. To delve further into that finding, we used a cell-based reporter assay to assess NOD1 activation through circulating agonists in Akita mice—a mouse model of type 1 diabetes. We observed that serum extracted from Akita mice 6 months after diabetes onset exhibited greater NOD1-activating potential than did serum extracted from age-matched controls (Fig. 1A). This finding suggests a higher presence of NOD1 activators in the circulation of type 1 diabetic mice.

### NOD1 depletion averted the decline in retinal thickness and the deterioration of retinal electrical responses induced by diabetes.

Male Akita mice serve as a well-established model of type 1 diabetes with discernible retinopathy characteristics [21, 22]. To further probe the involvement of NOD1 in the pathogenesis of DR, we generated NOD1^−/−^-Akita double-mutant mice and age-matched controls (Fig. 1B). The metabolic parameter values are shown in Fig. 1C–E. While both NOD1^−/−^-Akita and Akita mice had significantly higher glycated hemoglobin and glucose levels than did their nondiabetic control counterparts, NOD1 depletion did not increase glucose levels in diabetic mice. Akita mice exhibited reduced body weight relative to nondiabetic controls; however, no notable difference in body weight was observed between NOD1^−/−^-Akita and Akita mice.

**Fig. 2:**
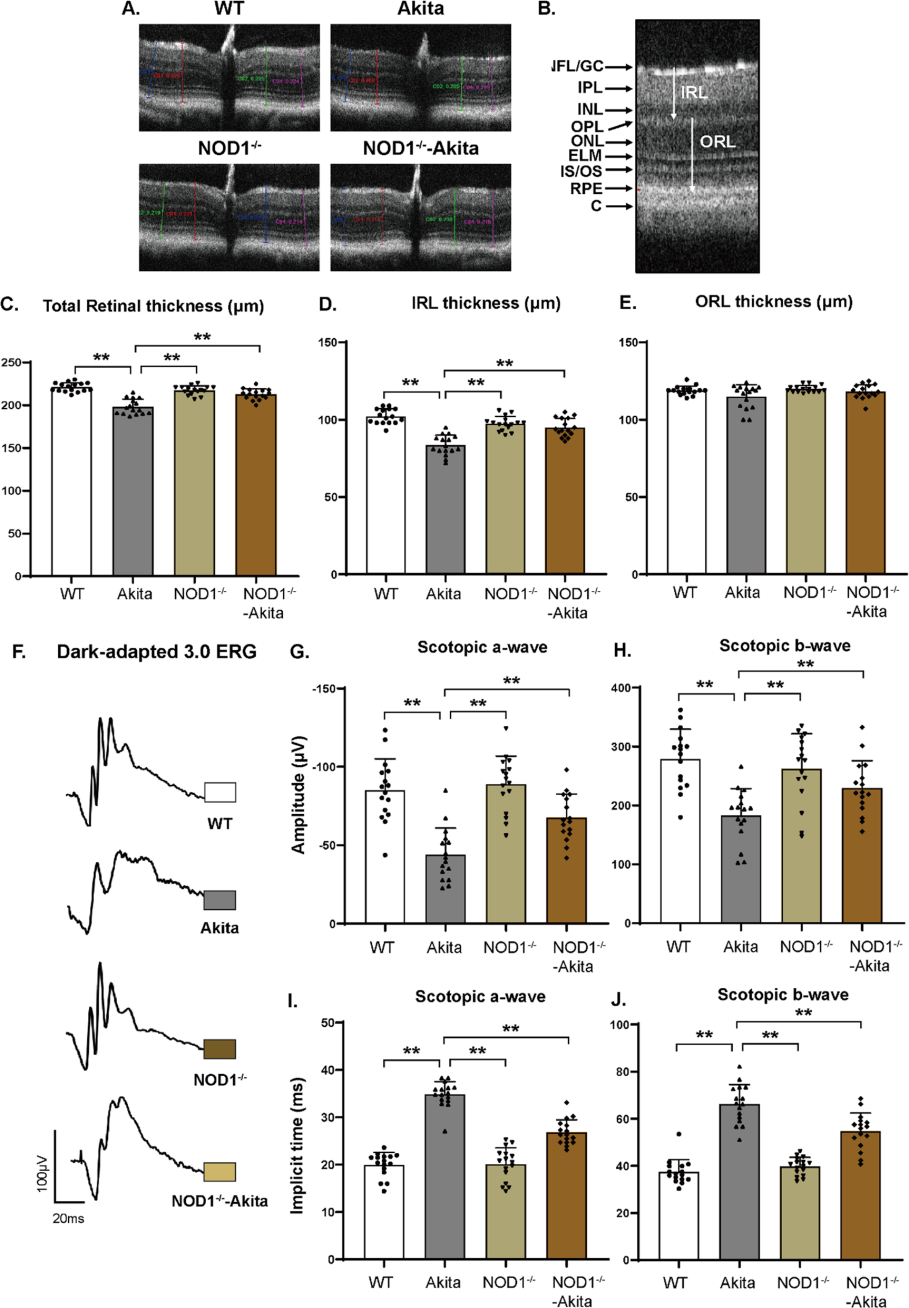
NOD1 knockout protects against diabetes-induced alterations in retinal morphology and electrical response. **A** Representative OCT images of retinal cross-sections. The distinct color lines indicate four measurements obtained at intervals of 150 and 300 µm from the optic disc. **B** Distinct intraretinal layers displayed by OCT. **C**–**E** Quantification of total retinal thickness, IRL and ORL by OCT (*n* = 16 per group). **F** Representative images of dark-adapted 3.0 ERG. **G**, **I** Amplitudes and implicit time of scotopic a-waves indicating electrical responses of rod photocurrents measured by ERG. **H**, **J** Amplitudes and implicit time of scotopic b-waves showing electrical signals of depolarizing bipolar cells and Müller cells measured by ERG (*n* = 16 per group). The results are presented as the mean ± SD; ^#^*p* < 0.05 compared with WT; ^§^*p* < 0.05 compared with Akita; **p < 0.01. WT, wild type; OCT, optical coherence tomography; ERG, electroretinogram; GC/NFL, ganglion/nerve fiber layer; IPL, inner plexiform; INL, inner nuclear layer; OPL, outer plexiform layer; ONL, outer nuclear layer; ELM external limiting membrane;IS/OS, photoreceptor inner/outer segments; RPE, retinal pigment epithelium; C, cone; IRL, inner retinal layer; ORL, outer retinal layer

Optical coherence tomography (OCT) is a noninvasive technique that captures cross-sectional retinal images and in vivo retinal changes. As depicted in Fig. 2A, C, retinal thickness diminished in Akita mice, with a 10% reduction compared to WT controls. The retinal thickness of NOD1^−/−^-Akita mice closely resembled that of WT and NOD1^−/−^ mice. This was also consistent with the findings of HE staining (Additional file 1: Fig. S1A–B). We further conducted an analysis of the inner retinal layer (IRL) and outer retinal layer (ORL) thicknesses (Fig. 2B). Our investigation unveiled that at 6 months of diabetes in Akita mice, alterations in total retinal thickness primarily stemmed from changes in the IRL (Fig. 2D). Conversely, no significant difference was noted in the thickness of the ORL (Fig. 2E). All the data indicate that NOD1 depletion partially resolved the structural alterations caused by diabetes in the cross-section of the retina.

Electroretinogram (ERG) analysis was used to assess the electrical responses of various retinal cell types to light stimulation and to evaluate diabetes-related visual function impairment [23]. At 6 months following diabetes onset, Akita mice exhibited diminished amplitudes and increased implicit time of both scotopic b-waves and photopic b-waves, reflecting reduced electrical responses of Müller cells and depolarizing bipolar cells (Fig. 2F–J, and Additional file 1: Fig. S2C–E). Notably, compared with Akita mice alone, NOD1^−/−^-Akita mice had augmented b-wave amplitudes under both scotopic and photopic conditions (Fig. 2H and Additional file 1: Fig. S2D). This suggests that NOD1 depletion improved retinal cell responses to light stimulation in diabetes. Additionally, the amplitude of the scotopic a-wave was lower in Akita mice than in WT controls (Fig. 2G). The partial restoration of the scotopic a-wave amplitude reduction in diabetic mice upon NOD1 depletion indicated that NOD1 depletion mitigated impaired rod photoreceptor activity (Fig. 2G). We also analyzed the oscillatory potentials (OPs), which are generated by inner retinal neurons, specifically amacrine cells. The amplitudes of OP a-wave and b-wave in Akita group were significantly decreased compared with those of the WT control group, while these changes were significantly reversed in the NOD1^−/−^-Akita group (Additional file 1: Fig. S1F–L).

### Loss of NOD1 attenuated the progression of diabetic retinal vascular degeneration

**Fig. 3:**
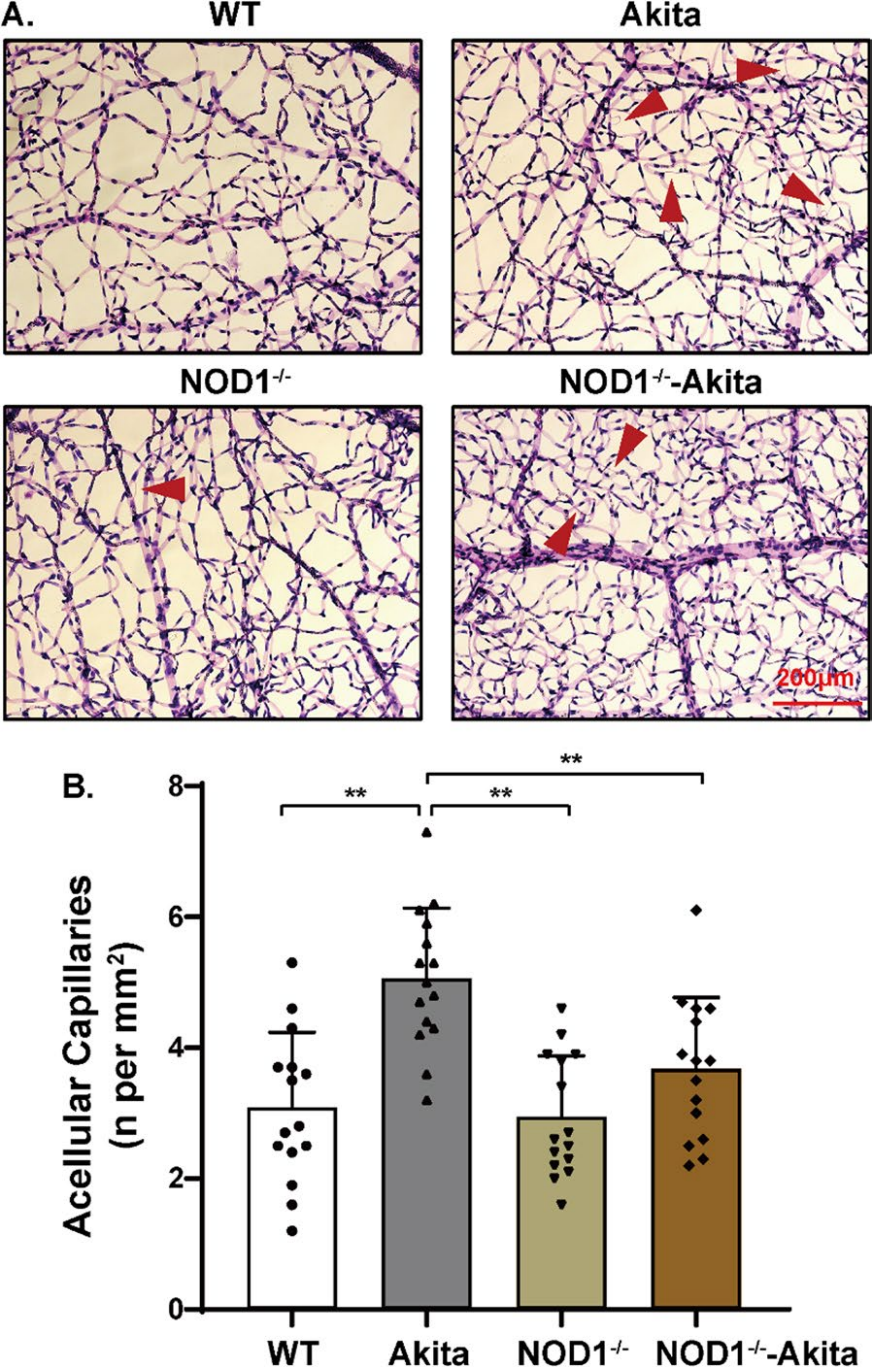
NOD1 ablation slows diabetic retinal vascular degeneration. **A** Retinal vasculature stained with PAS-hematoxylin showing acellular capillaries (red arrows), a hallmark of DR. **B** Quantification of acellular capillaries shown in the bottom panel (*n* = 15 per group). The results are presented as the mean ± SD; **p < 0.01. WT, wild type

Microvascular degeneration is a prominent pathological hallmark of DR. The quantification of acellular capillaries, i.e., basal membrane tubes devoid of nuclei, serves as the gold standard for evaluating DR in animal models [24]. Compared with WT controls, Akita mice 6 months after diabetes onset exhibited a notable increase in the number of acellular capillaries (Fig. 3A, B). Intriguingly, the absence of NOD1 significantly mitigated microvascular impairment within the diabetic retina (Fig. 3A, B), thus underscoring the pivotal role of NOD1 in the progression of DR.

### The absence of NOD1 rectified the diabetes-induced imbalance in HSPC subpopulations

**Fig. 4:**
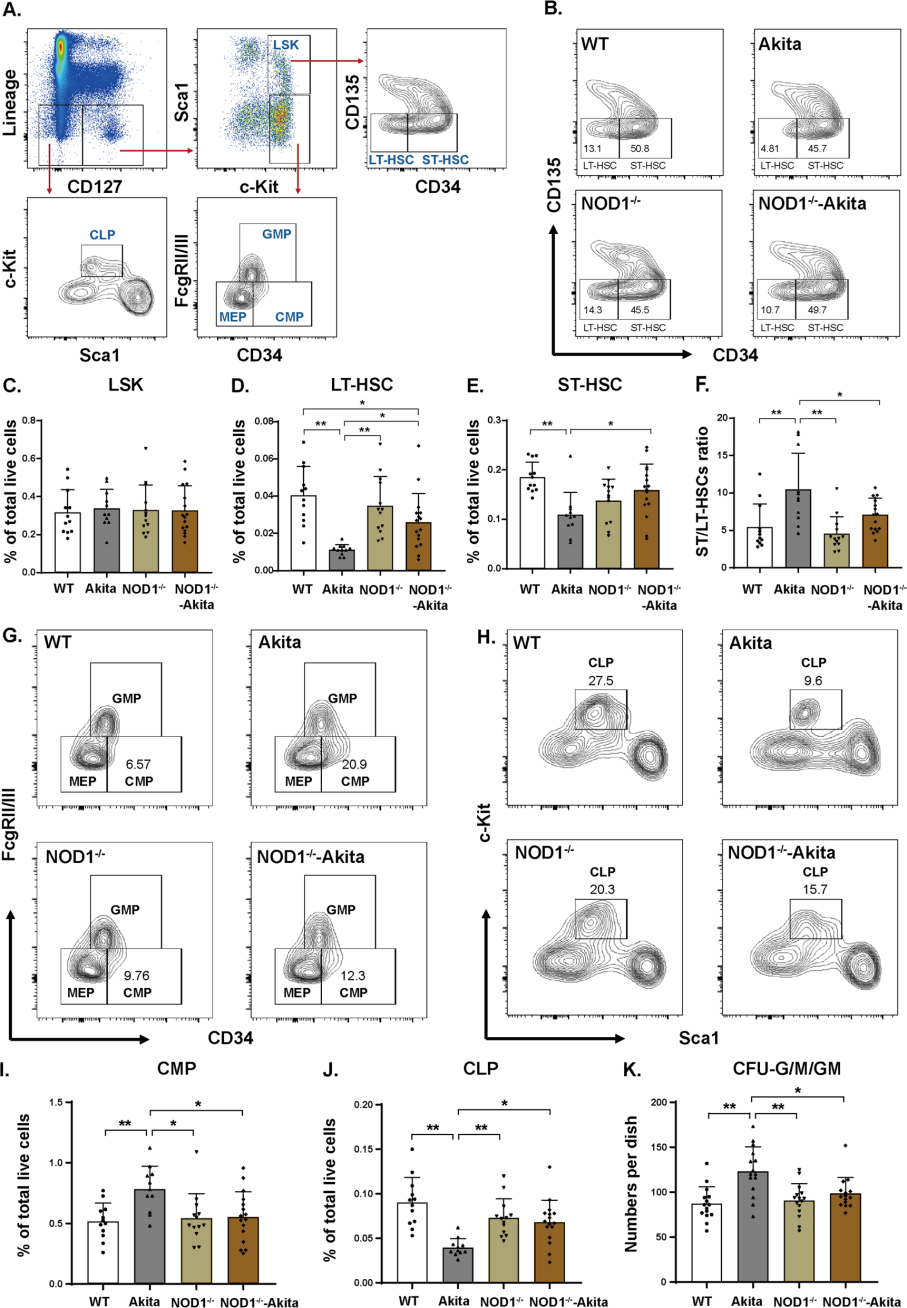
NOD1 depletion corrects diabetes-induced hematopoietic imbalance. **A** Flow cytometry gating strategy for bone marrow single-cell suspensions. Viable/live singlets were gated on Lin^−^CD127^±^ cells. Representative FACS plots of the analysis of HSPC subpopulations gated as LSK (Lin^−^CD127^−^/Sca1^+^c-Kit^+^), LT-HSC (Lin^−^CD127^−^/Sca1^+^c-Kit^+^/CD34^−^CD135^−^), ST-HSC (Lin^−^CD127^−^/Sca1^+^c-Kit^+^/CD34^+^CD135^−^), CLP (Lin^−^CD127^−^/Sca1^med^c-Kit^med^), CMP (Lin^−^CD127^−^Sca1^−^c-Kit^+^CD34^+^FcgRII/III^lo^), MEP (Lin^−^CD127^−^Sca1^−^c-Kit^+^CD34^−^FcgRII/III^lo^) and GMP (Lin^−^CD127^−^Sca1^−^c-Kit^+^CD34^+^FcgRII/III^hi^) (*n* = 11–16 per group). **B** Representative flow cytometry diagrams of LT- and ST-HSCs in each experimental group. **C**–**E** Percentages of bone marrow LSK, LT-HSCs and ST-HSCs out of total live cells. **F** The ratio of the percentages of ST-HSCs to LT-HSCs in bone marrow. **G**, **H** Representative flow cytometry diagrams of CMP and CLP cells in each experimental group. **I**–**J** Percentages of bone marrow CMP and CLP cells out of total live cells. **K** CFU assay results, i.e., quantification of the numbers of CFU-G/M/GMs after plating bone marrow cells. (*n* = 15 per group). The results are presented as the mean ± SD; *p < 0.05, **p < 0.01. WT, wild type; HSPC, hematopoietic stem/progenitor cell; LT-HSC, long-term repopulating hematopoietic stem cell; ST-HSC, short-term repopulating hematopoietic stem cell; CLP, common lymphoid progenitor; CMP, common myeloid progenitor; GMP, granulocyte–macrophage progenitor; MEP, megakaryocyte erythroid progenitor

In a prior study, we elucidated how the NOD1 ligand disrupts the equilibrium and vasoreparative functionalities of hematopoietic stem/progenitor cells (HSPCs) within diabetic bone marrow [8]. In this context, we explored whether NOD1-depletion impediment of DR progression is connected to modifications in the bone marrow phenotype. Compared with WT mice, Akita mice 6 months after diabetes onset displayed a reduction in the percentage of both short-term (ST)- and long-term (LT)-repopulating hematopoietic stem cells (HSCs) in the bone marrow, as determined by flow cytometry (Fig. 4A–E). Additionally, the ratio of ST-HSCs to LT-HSCs was higher in Akita mice than in WT mice (Fig. 4F), indicating an irregularity in HSC subpopulations and a disruption in hematopoiesis within the diabetic bone marrow. The bone marrow of NOD1^−/−^-Akita mice, compared with that of Akita mice alone, exhibited partial restoration in the percentages of ST-HSCs and LT-HSCs (Fig. 4D, E). Furthermore, we conducted a detailed flow cytometric analysis of bone marrow hematopoiesis across the four cohorts. Interestingly, we noted that in contrast to bone marrow from the WT group, although diabetic bone marrow had a higher percentage of CMPs and a lower percentage of CLPs, NOD1 depletion reinstated the equilibrium of hematopoiesis in the diabetic milieu (Fig. 4G–J). Consistent with the flow cytometry findings, the CFU assay also revealed elevated numbers of CFU-G/M/GMs in the bone marrow of Akita mice, signifying a shift toward a more proinflammatory hematopoietic state in diabetes (Fig. 4K). Notably, the double-mutant mice exhibited a CFU colony-forming capacity akin to that of the WT group, underscoring that NOD1 depletion restored the diabetes-induced hematopoietic imbalance toward myelopoiesis.

### NOD1 chimeras slowed the progression of DR

**Fig. 5:**
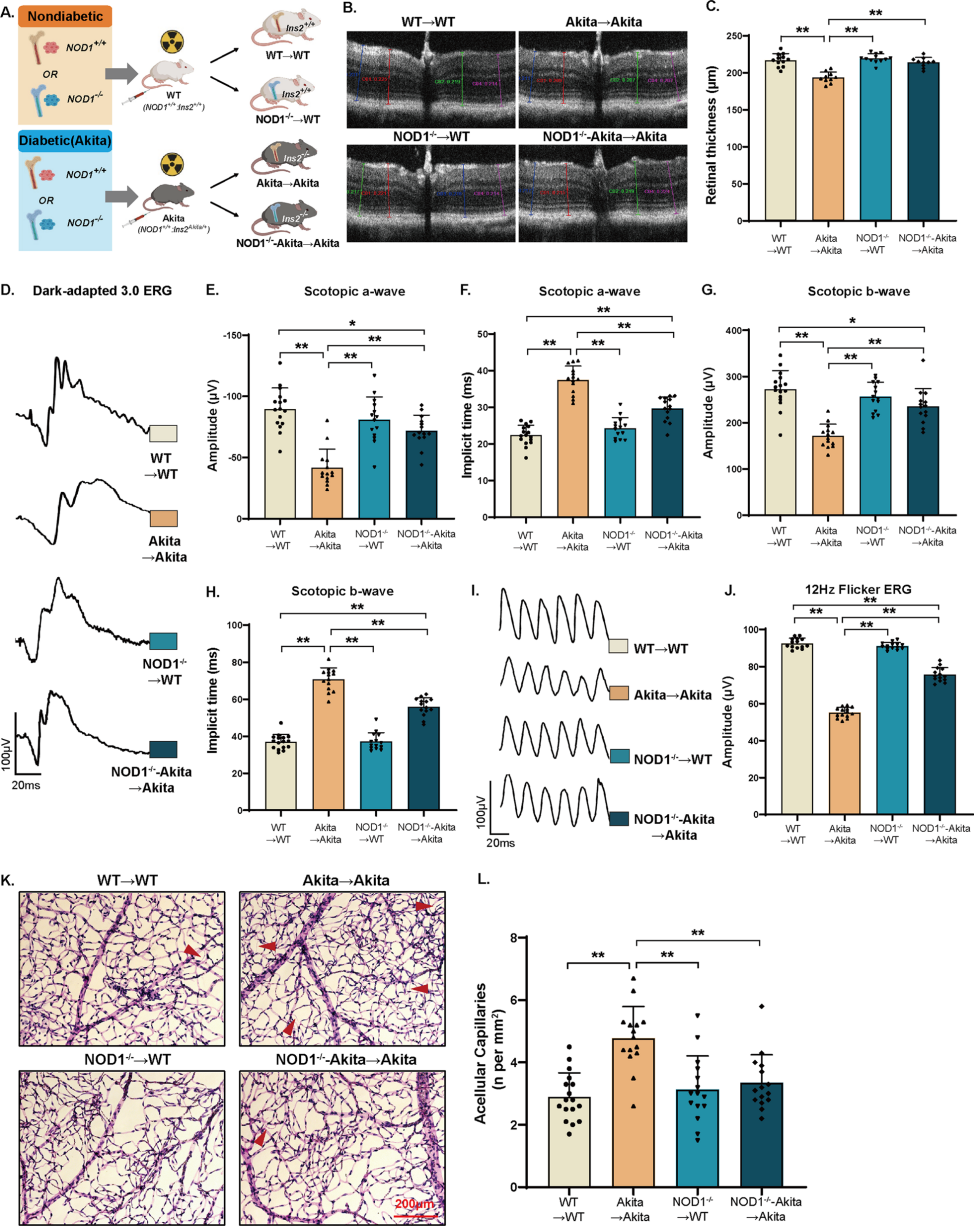
Hematopoietic-specific NOD1 knockout ameliorates the progression of DR. **A** Study design for bone marrow transplantation. **B** Representative OCT images of retinal cross-sections (*n* = 10–12 per group). **C** Bar plots showing the quantification of retinal thickness of mice from diabetic and nondiabetic NOD1 chimeras as well as their controls. **D** Representative scotopic ERG waveforms from the chimera mice. **E**–**H** ERG amplitudes and implicit times of scotopic a-waves and b-waves (*n* = 14–16 per group). **I** Representative 12-Hz-flicker ERG waveforms. **J** The amplitudes of 12-Hz-flicker ERG in each group (*n* = 14 per group). **K** Retina vasculature was obtained through trypsin digestion and subsequently stained with PAS-hematoxylin. The presence of acellular capillaries, indicated by red arrows, is a characteristic pathological hallmark of DR. **L** Quantification of acellular capillaries in the respective groups (*n* = 15–17 per group). Data are presented as the mean ± SD; *p < 0.05, **p < 0.01. WT, wild type; OCT, optical coherence tomography; ERG, electroretinogram

To further investigate the role of hematopoietic NOD1 in DR development, we conducted bone marrow transplantation to create mice with hematopoietic cell-specific NOD1 deficiency (Fig. 5A). OCT revealed a notable 10.9% reduction in retinal thickness within the Akita → Akita group compared to the nondiabetic controls (Fig. 5B, C). The specific loss of NOD1 in hematopoietic cells mitigated the structural changes in retinal cross-sections caused by diabetes (Fig. 5B, C). These alterations were also validated by H&E staining (Additional file 1: Fig. S2A–D). On ERG, compared with the Akita → Akita group, the NOD1^−/−^-Akita → Akita group exhibited enhanced scotopic a-wave and b-wave responses as well as photopic b-wave responses (Fig. 5D, H and Additional file 1: Fig. S3A–C). This suggests that the absence of bone marrow NOD1 restored the electrical responses of the retina to light stimuli. Moreover, the quantification of acellular capillaries, a hallmark of vascular degeneration in DR, revealed that hematopoietic NOD1 ablation alleviated the diabetes-induced increase in this pathological feature (Fig. 5K, L). In the analysis of OPs, the amplitudes of the a-wave and b-wave in the NOD1^−/−^-Akita → Akita group showed a significant increase compared to those of the Akita → Akita group (Additional file 1: Fig. S3D–J). Flicker ERG, reflecting changes in microvascular circulation in diabetic retinopathy, displayed representative waveforms in Fig. 5I. The NOD1^−/−^-Akita → Akita group exhibited significantly higher amplitudes compared to the Akita → Akita group. Consequently, flicker ERG responses increased with NOD1 depletion in Akita mice, while WT → WT and NOD1^−/−^ → WT responses remained unchanged (Fig. 5J).

### Hematopoietic-specific NOD1 depletion led to the reduced infiltration of proinflammatory cell types in the diabetic retina

**Fig. 6:**
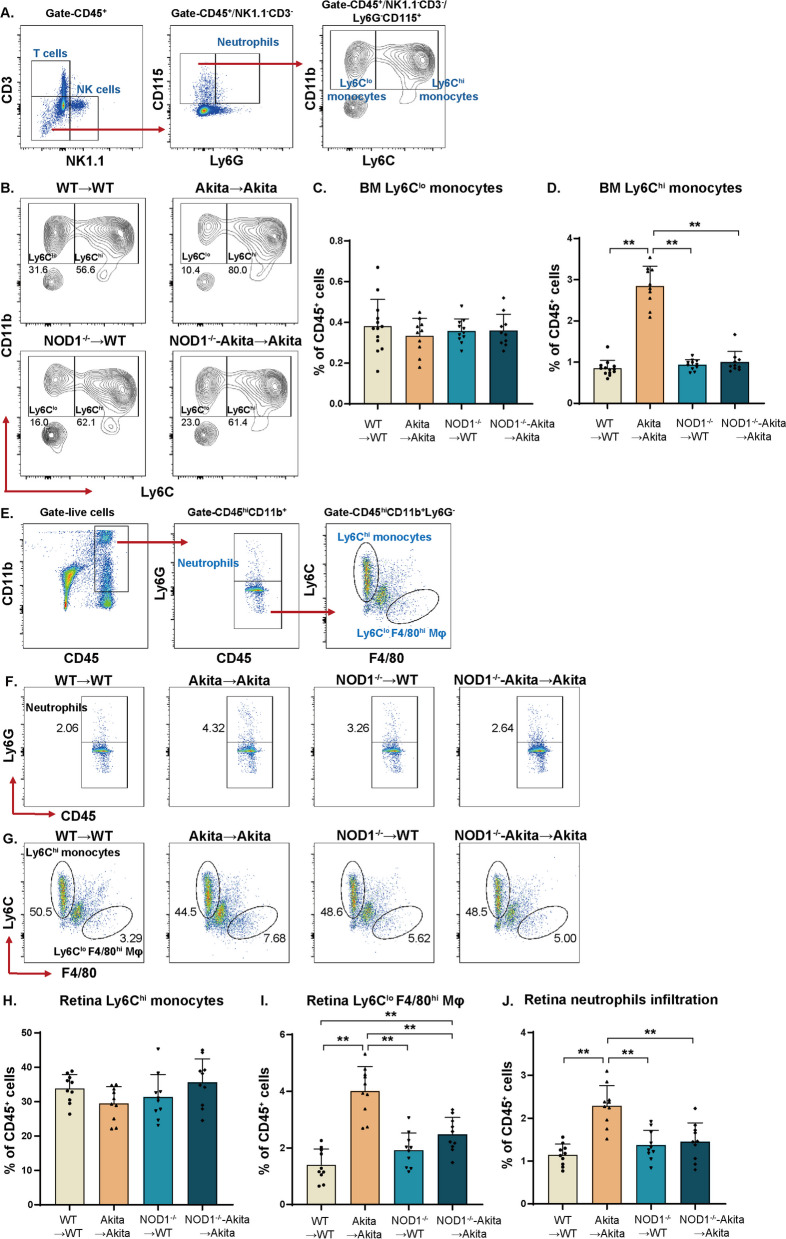
Reduced infiltration of bone marrow-derived macrophages in the retina of diabetic NOD1 chimeras. **A** Flow cytometry gating strategy for Ly6C^lo^ and Ly6C^hi^ monocytes in bone marrow. **B** Flow cytometry diagrams of bone marrow monocytes from WT → WT, NOD1^−/−^ → WT, Akita → Akita and NOD1^−/−^-Akita → Akita mice. **C**, **D** The percentages of Ly6C^lo^ and Ly6C^hi^ monocytes among CD45^+^ cells (flow cytometry analysis; *n* = 10–13 per group). **E** Flow cytometry gating strategy for bone marrow-derived monocytes (CD45^hi^CD11b^+^Ly6G^−^Ly6C^hi^), macrophages (CD45^hi^CD11b^+^Ly6G^−^Ly6C^lo^F4/80^hi^) and neutrophils (CD45^hi^CD11b^+^Ly6G^+^) in pooled retina samples from the respective groups. **F** Representative images of neutrophils in the retina. **G** Ly6C^hi^ monocytes and Ly6C^lo^F4/80^hi^ macrophages in the retina. **H**–**J** Bar graphs showing the percentages of retinal Ly6C^hi^ monocytes, Ly6C^lo^F4/80^hi^ macrophages and neutrophils among CD45^+^ cells (*n* = 10 per group). Data are presented as the mean ± SD; *p < 0.05, **p < 0.01. WT, wild type; BM, bone marrow; MΦ, macrophage

To elucidate the mechanisms underlying DR progression resulting from the specific loss of NOD1 in hematopoietic cells, we conducted a flow cytometric analysis of myeloid populations in both bone marrow and retinas. The gating strategy for bone marrow monocyte populations is outlined in Fig. 6A. Among the four cohorts, no significant alteration was observed in the Ly6C^lo^ monocyte population (Fig. 6B, C). Nonetheless, the number of proinflammatory Ly6C^hi^ monocytes was substantially higher in the bone marrow of Akita → Akita mice than in the bone marrow of WT → WT control mice. However, after hematopoietic NOD1 loss in Akita mice, this pathological shift was prevented (Fig. 6D).

Monocytic cells have the capacity to infiltrate the retina and differentiate into monocyte-derived macrophages, which reside in the retina over time and contribute to retinal vascular degeneration through paracrine mechanisms. The gating schema for retinal cells, as illustrated in Fig. 6E, differentiated bone marrow-derived monocytes, macrophages, and infiltrating neutrophils [19, 20]. The percentage of bone marrow-derived Ly6C^hi^ monocytes was consistent between retinas from WT → WT control mice and Akita → Akita mice (Fig. 6G, H). Notably, the accumulation of monocyte-derived macrophages in the retina increased from 1.4% of total CD45^+^ cells in WT → WT mice to 4.0% in the Akita → Akita group (Fig. 6I). Hematopoietic NOD1 loss had a protective effect against diabetes, leading to fewer monocyte-derived macrophages residing in the retina of NOD1^−/−−^-Akita → Akita mice (Fig. 6I). Furthermore, alterations in macrophage profiles associated with NOD1 depletion were correlated with a reduction in neutrophil infiltration in the retina of NOD1^−/−^-Akita → Akita mice; the same was not observed for the Akita → Akita group (Fig. 6F, J).

### NOD1-mediated paracrine effects of bone marrow-derived macrophages drive neutrophil infiltration and NETosis

**Fig. 7:**
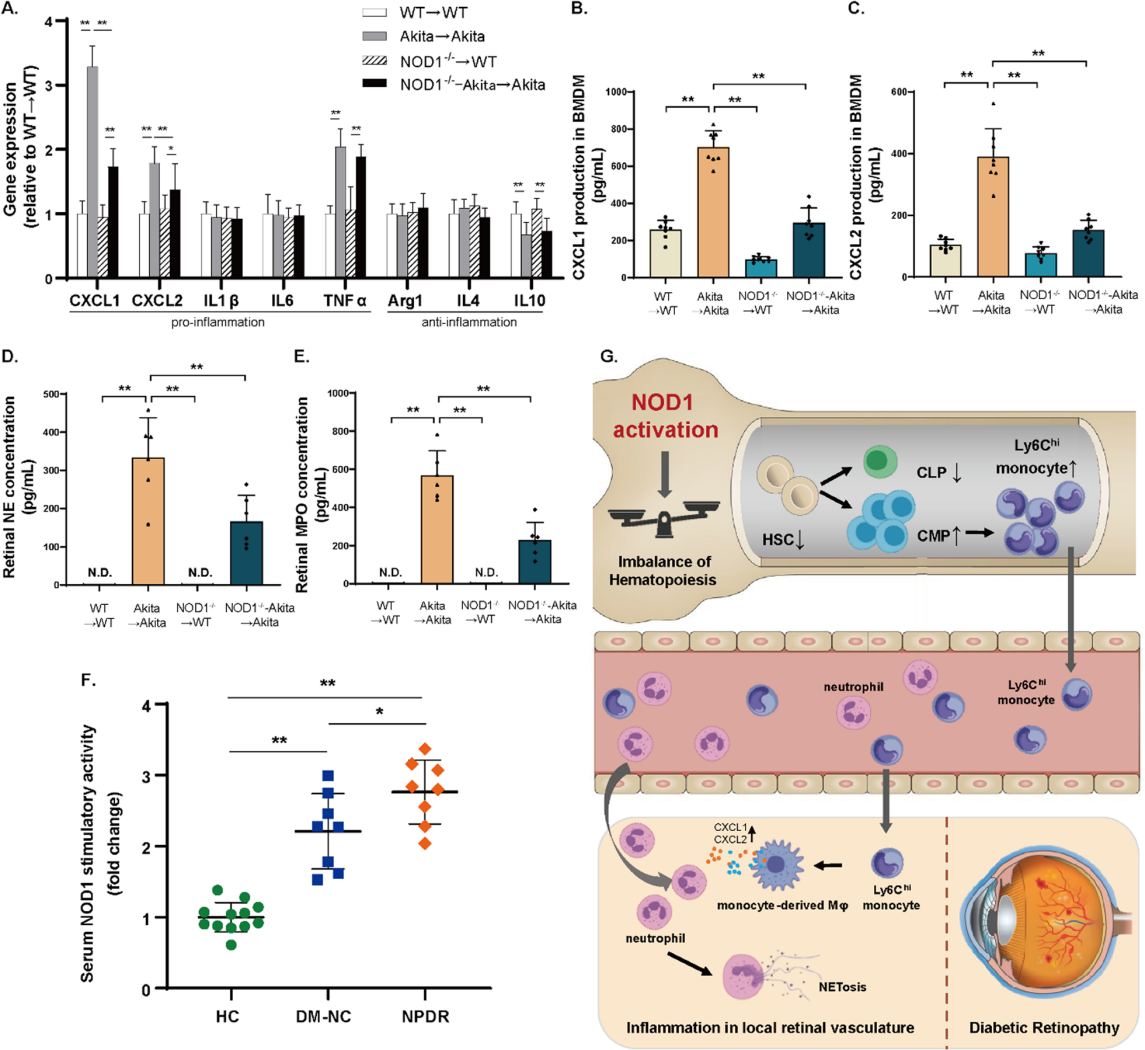
Hematopoietic NOD1 deficiency mitigates the increase in neutrophil chemoattractants and NETosis markers in Akita mice. **A** Quantitative PCR analysis of proinflammatory and anti-inflammatory gene expression in the retinas of NOD1^−/−^-Akita → Akita mice and their cohorts (*n* = 8 per group). **B**, **C** Measurement of CXCL1 and CXCL2 production in the culture supernatant of BMDMs treated with peptidoglycan for 24 h (ELISA, *n* = 8 per group). **D**, **E** Detection of neutrophil elastase and myeloperoxidase in the retina (ELISA, *n* = 6 per group). **F** Assessment of NOD1 stimulatory activity in serum samples from human subjects using a HEK293T cell-based reporter assay (HC, *n* = 12; DM-NC, *n* = 8; NPDR, *n* = 8). **G** Schematic diagram of the mechanisms of hematopoietic NOD1-mediated DR progression. Data are presented as the mean ± SD; *p < 0.05, **p < 0.01. WT, wild type; N.D., nondetectable; NE, neutrophil elastase; MPO, myeloperoxidase; HC, healthy controls; DM-NC, diabetes with no microvascular complications; NPDR, nonproliferative diabetic retinopathy

Given the influence of NOD1 on myeloid cell infiltration in diabetic retinas, we delved deeper into functional gene expression, including the expression of both proinflammatory (CXCL1, CXCL2, IL1β, IL6, TNFα) and anti-inflammatory (Arg1, IL4, IL10) genes. As seen in Fig. 7A, in diabetic retinas, mRNA levels of CXCL1, CXCL2, and TNFα were increased, and IL10 levels were decreased. Intriguingly, the hematopoietic depletion of NOD1 mitigated the diabetes-induced expression of CXCL1 and CXCL2; TNFα and IL10 remained unaffected. These chemoattractants, CXCL1 and CXCL2, are chiefly secreted by bone marrow-derived macrophages (BMDMs) and are pivotal for neutrophil recruitment and activation. Correspondingly, PGN stimulation led to a substantial increase in CXCL1 and CXCL2 protein secretion by diabetic BMDMs ex vivo, an effect that was counteracted by NOD1 deficiency (Fig. 7B, C). Notably, the expression of markers for NETosis, myeloperoxidase (MPO), and neutrophil elastase (NE) increased significantly in diabetic retinas, suggesting that chemoattractant-driven neutrophil recruitment could be connected to heightened NETosis in DR. However, these NETosis markers were markedly decreased in retinas isolated from NOD1^−/−^-Akita → Akita mice (Fig. 7D, E). Collectively, these findings imply that hematopoietic NOD1 significantly influences BMDM activation and chemoattractant production, triggering neutrophil infiltration and NETosis, which could worsen local inflammation and exacerbate DR progression.

To offer further insight into the role of NOD1 in DR, we recruited diabetic subjects either without microvascular complications or with DR as well as age- and sex-matched healthy controls. Serum samples were collected to assess NOD1 activity through a cell-based reporter assay. As anticipated, circulating NOD1 activity was higher in patients with diabetes without microvascular complications than in healthy controls. In patients with DR, serum NOD1 stimulatory activity was high, corroborating the pivotal role of NOD1 in DR (Fig. 7F). Targeting NOD1 could hold promise as a strategy for predicting and intervening in DR progression.

## Discussion

In this study, we unveiled the pivotal role of NOD1 as a critical mediator in regulating bone marrow–retina interorgan communication, which significantly impacts the development of DR (Fig. 7G). Our findings, consistent across both mouse models and human subjects with DR, indicated elevated levels of circulating NOD1 activators. NOD1 depletion effectively counteracts the diabetes-induced depletion of both LT- and ST-HSCs as well as disruptions in hematopoiesis, redirecting cells toward a more proinflammatory profile. Remarkably, we established that specifically silencing NOD1 within the hematopoietic system results in reduced BMDM infiltration and chemoattractant production in the retina. This, in turn, diminishes neutrophil recruitment and NETosis, ultimately slowing the progression of DR from a mechanistic standpoint.

A growing body of evidence underscores the profound impact of diabetes- and obesity-induced microbiota alterations on metabolism and immunity [25, 26]. Pathogenic bacteria furnish an array of PAMPs, including LPS and PGN, which enter the bloodstream due to compromised gut epithelial and vascular barriers. These PAMPs subsequently engage pattern recognition receptors (PRRs), setting off a state of low-grade inflammation and initiating innate immunity [10, 27].

NOD1, a prominent cytoplasmic PRR, plays a central role in sensing specific PAMP and PGN fragments [26]. Its association with insulin resistance, a hallmark of type 2 diabetes, has come into focus. Accumulating evidence suggests that NOD1 activation triggers the NF-κB and MAPK pathways, facilitating insulin resistance in the liver and adipocytes [26, 28]. This results in reduced glucose uptake, heightened hepatic gluconeogenesis, and ultimately elevated blood glucose levels in mouse models of obesity and type 2 diabetes [10, 29, 30]. However, the relevance of NOD1 to type 1 diabetes and diabetic vascular complications has remained relatively unexplored. Using the Akita mouse model, our investigation revealed a pivotal role for NOD1 in the progression of DR within the context of type 1 diabetes. Remarkably, this effect is observed without discernible impacts on blood glucose levels or body weight. Notably, NOD1 depletion confers protective effects across several facets of DR pathogenesis, spanning alterations in retinal thickness, the attenuation of light-induced electrical responses, and the mitigation of retinal vasculature damage. These findings usher in a new dimension of understanding regarding the role of NOD1 in diabetes.

The pathobiological processes contributing to the development of diabetic retinopathy (DR) are multifaceted and involve intricate cellular and molecular mechanisms. These mechanisms encompass hyperglycemia-triggered oxidative stress, inflammation, PKC pathways, microbiota alterations, and imbalances in HSPCs [31–33]. Among these, NOD-like receptors (NLRs), including NOD1, NLRP1, and NLRP3, serve as vital interfaces between microbiota and systemic chronic inflammation. Notably, NLRP1 deficiency has been shown to mitigate retinal abnormalities induced by diabetes in a streptozotocin-induced diabetic mouse model [34]. Additionally, another study highlighted the protective effects of fenofibrate administration against retinal vascular leakage through the regulation of NLPR2 signaling and a reduction in NLPR3 activation [35]. Our investigation unveiled a significant yet partial attenuation of retinal pathological changes in the Akita mouse model upon NOD1 ablation. This discovery establishes that NOD1, another member of the NLR family, plays a pivotal role in the progression of DR. Importantly, this finding alludes to the potential involvement and synergistic impacts of multiple NLRs. Moreover, it underscores the possibility that various member of the NLR family could offer novel therapeutic avenues for addressing DR treatment.

The significance of an imbalanced state of HSPCs in DR is widely recognized. Previous research, including our own and others', has consistently highlighted various detrimental changes that occur in the bone marrow during DR. These alterations include diminished HSPC numbers, impaired vasoreparative functions of these cells, and an increased shift toward myelopoiesis and proinflammatory cell types [8, 17, 22, 36]. NOD1, as a pivotal player within the innate immune system, is notable because of its capacity to discern pathogens and subsequently induce the production of myeloid lineage cells within the bone marrow. This process is critical for the release of cytokines and the initiation of inflammatory responses [25, 37]. Iwamura et al. revealed that administering a NOD1 ligand restored the pool of hematopoietic stem cells and their precursors within the bone marrow of germ-free mice [13]. Another study illuminated the role of hematopoietic-specific NOD1 in regulating the infiltration of neutrophils and proinflammatory macrophages within adipose tissue in mice fed a high-fat diet [10]. In our current investigation, we also established that NOD1 depletion counteracted the diabetes-induced reduction in HSC populations and mitigated the shift toward myelopoiesis. This observation is further substantiated by the relief of diabetic retinopathy phenotypes through the reduced infiltration of bone marrow-derived macrophages in hematopoietic-specific NOD1^−/−^-Akita mice. These findings support NOD1 as a significant mediator within the "bone marrow-retina" axis in the context of DR.

A key finding of this study is that NOD1 plays an important role in mediating the infiltration of bone marrow-derived proinflammatory cells, such as neutrophils and monocyte-derived macrophages, in the diabetic retina. The results of studies by others and by us support the idea that when systemic inflammation becomes more pronounced, circulating inflammatory cells can infiltrate the retina, exacerbating the local inflammatory response and contributing to vascular dysfunction [11]. The role of these circulating cells may extend beyond the local environment, reflecting the systemic nature of inflammation in diabetic retinopathy. Notably, in addition to circulating cells, resident inflammatory cells, such as microglia and Müller cells, play pivotal roles in the local inflammatory milieu of the diabetic retina [38]. These cells are resident immune cells with the potential to respond swiftly to changes in the microenvironment. The interaction and crosstalk between resident and circulating inflammatory cells are likely integral to the progression of diabetic retinopathy. Resident cells may act as initiators, responding to early insults, and circulating cells may perpetuate and amplify the inflammatory cascade as the disease advances [38]. Further research is needed to elucidate the molecular and cellular interactions between these two components of the immune system, potentially providing valuable insights into the intricate mechanisms underlying disease progression. Importantly, NOD1 is extensively expressed in local retinal tissue [39], prompting the necessity for further studies to gain a comprehensive understanding of its precise role and the intricate interplay between retinal NOD1 and NOD1 within the hematopoietic system.

A particular discovery that emerged from our study pertains to the balance of Ly6C^hi^ monocytes within the bone marrow and their corresponding proinflammatory monocyte population in the retina of Akita mice. Despite observing an increase in the number of Ly6C^hi^ monocytes within the bone marrow of Akita mice, we noted that the number of proinflammatory monocytes derived from the bone marrow in the retina remained unchanged. One plausible interpretation of this observation is that upon migration into the retina, a considerable fraction of these monocytes undergo differentiation into macrophages [40]. These macrophages, in turn, intensify the inflammatory response by releasing an array of cytokines and other inflammatory molecules. Our data reinforce this hypothesis, as we observed marked infiltration of BMDMs into the retinas of Akita mice. A compelling aspect is that the deficiency of hematopoietic NOD1 alone achieved through bone marrow transplantation is sufficient to substantially diminish the presence of these macrophages within the retinas of diabetic mice.

Inflammatory molecule cascades can further damage blood vessels within the retina, ultimately contributing to the emergence of microvascular degeneration, a well-recognized hallmark of DR. Our in vivo and ex vivo data consistently demonstrate that BMDMs within the diabetic retina produce excessive amounts of CXCL1 and CXCL2. These chemokines are potent chemoattractants responsible for attracting neutrophils to the site of inflammation [41, 42]. This finding aligns with the results of a recent study in which there were elevated levels of CXCL1 in the retinas of individuals with diabetic retinopathy [43]. Notably, the intravitreal administration of CXCL1 led to increased retinal vascular permeability accompanied by neutrophil recruitment [43].

Notably, we observed that NOD1 closely participates in the regulation of CXCL1 and CXCL2 production within the diabetic retina. Intriguingly, we found that NOD1 depletion in hematopoietic cells does not exert any influence on diabetes-induced alterations in TNFα and IL10, underscoring the intricate nature of the inflammatory response operating within the diabetic retina. This interplay of NOD1 and these chemokines signifies their pivotal role in driving retinal inflammation and further emphasizes the complexity of the mechanisms at play in DR.

The role of NETosis, a specific process involving neutrophil cell death, has garnered attention with regard to understanding the development of DR [44, 45]. Studies have shown increased neutrophil extracellular trap (NET) levels in the serum and retinas of diabetic individuals, possibly due to NADPH oxidase activation [44]. NETosis, which plays a role in removing senescent vasculature, may contribute to vascular remodeling and angiogenesis in DR [45]. Additionally, research has found that knocking out the NETosis marker NE protects retinal vessels from leakage and degeneration in the early stages of DR [46]. In our study, we discovered that NOD1 activation in hematopoietic cells leads to the production of the NETosis markers NE and MPO as well as the recruitment of neutrophils to the retinal microenvironment. These findings indicate that NOD1 plays a pivotal role in orchestrating this intricate sequence of events. By regulating the infiltration of BMDMs, the production of chemoattractants, and subsequent neutrophil recruitment, NOD1 activation contributes to the NETosis-driven inflammation observed in diabetic retinopathy. Additional research is needed to comprehensively grasp the intricate and detailed mechanisms governing the regulation of the NETosis process in the context of DR.

## Conclusions

Our study sheds light on the involvement of NOD1 in hematopoietic imbalance leading to local retinal inflammation and the progression of diabetic retinopathy. These findings suggest a potential role for NOD1 as a therapeutic target. However, further studies are warranted to comprehensively assess the safety profile and potential systemic effects of targeting NOD1 in bone marrow for the prevention and treatment of diabetic retinopathy.

### Supplementary Information

**Additional file 1**. Supplementary data.

## Data Availability

All data generated or analyzed during this study are included in this published article. Data generated during the current study are available from the corresponding author upon reasonable request.

## Abbreviations

NOD1: Nucleotide-binding oligomerization domain-containing protein 1
DR: Diabetic retinopathy
NPDR: Nonproliferative diabetic retinopathy
PGN: Peptidoglycan
LPS: Lipopolysaccharide
PAMP: Pathogen-associated molecular pattern
TLR4: Toll-like receptor 4
NE: Neutrophil elastase
MPO: Myeloperoxidase
NET: Neutrophil extracellular trap
HSPC: Hematopoietic stem/progenitor cell
HSC: Hematopoietic stem cells
BMDM: Bone marrow-derived macrophage
CLP: Common lymphoid progenitor
CMP: Common myeloid progenitor
GMP: Granulocyte–macrophage progenitor
MEP: Megakaryocyte erythroid progenitor
OCT: Optical coherence tomography
ERG: Electroretinography
