# Supplementary material for: NOD1 deficiency ameliorates the progression of diabetic retinopathy by modulating bone marrow–retina crosstalk
Source: Stem Cell Res Ther. 2024 Feb 9;15:38. doi: 10.1186/s13287-024-03654-y (PMC10858517; doi:10.1186/s13287-024-03654-y)
Supplement: Supplementary file 1 — Additional file 1. Supplementary data. [file 13287_2024_3654_MOESM1_ESM.docx]

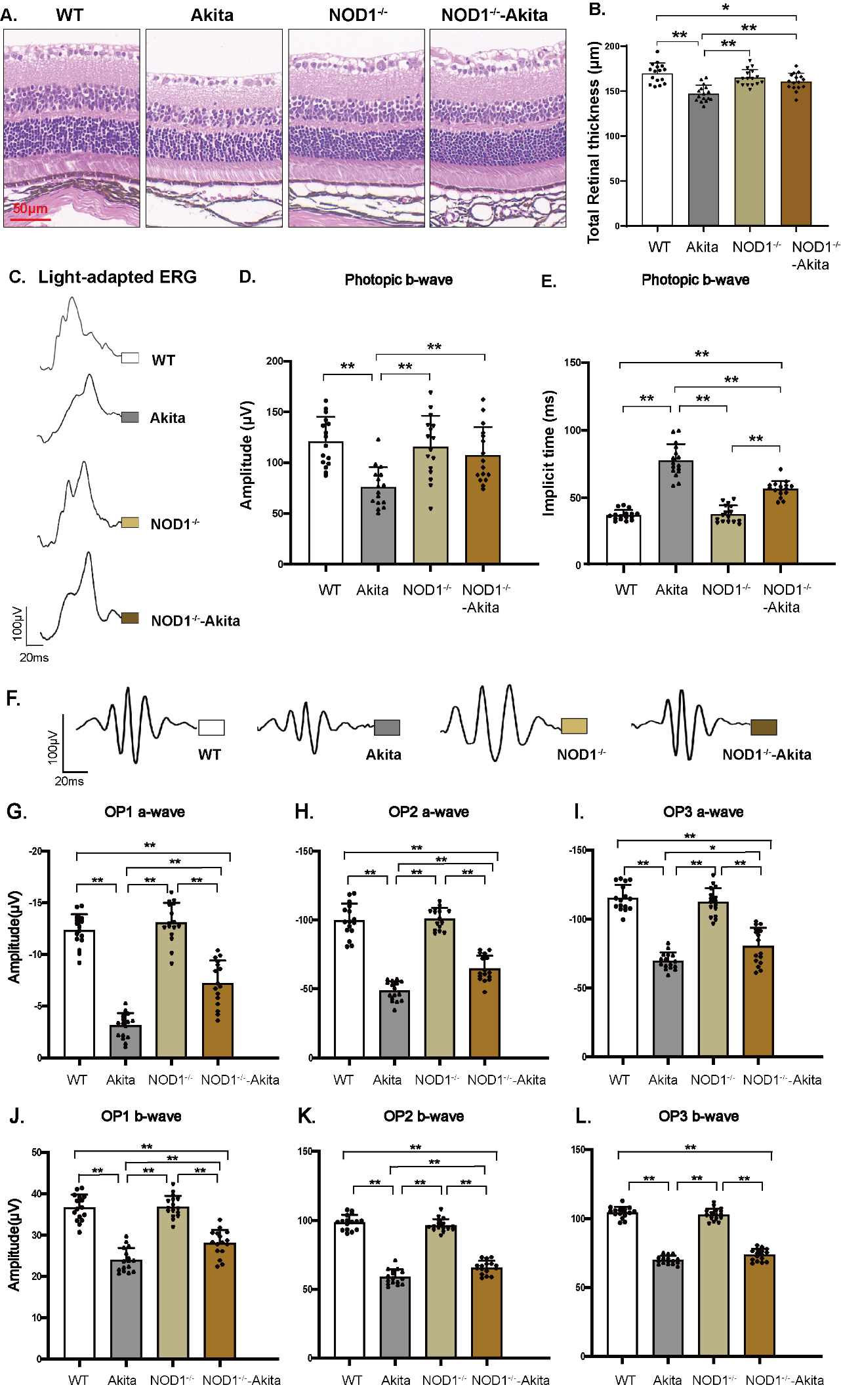


**Supplementary Figure 1: ERG and Retinal Histological Changes in NOD1 Knockout Diabetic and Nondiabetic Mice.** (A) Representative retinal hematoxylin-eosin (HE) staining of four different groups. (B) Quantification of retinal thickness by HE staining (n=16 per group). (C) Representative photopic ERG waveforms generated for each group with a flash intensity of 3.0 cd·s/m2 after light adaptation for 10 minutes. Photopic b-wave amplitude and implicit time were measured by ERG (n=16 per group). (D) Comparison of photopic b-wave amplitudes in each group. (E) Differences in the photopic b-wave implicit times in the four groups. Oscillatory potentials (OPs) were marked, with the first peak following the a-wave lowest points identified as OP1, and subsequent peaks as OP2 and OP3. (F) Representative ERG OPs waveforms from WT, diabetic Akita, and NOD1 knockout mice are shown. (G-L) Changes in the amplitude of a-wave and b-wave for OP1, OP2, OP3 in different groups (n=16 per group). Results are shown as mean ± SD; *, p<0.05, **, p<0.01. Abbreviations: HE, hematoxylin-eosin staining; WT, wild type; ERG, electroretinogram; OPs, oscillatory potentials.


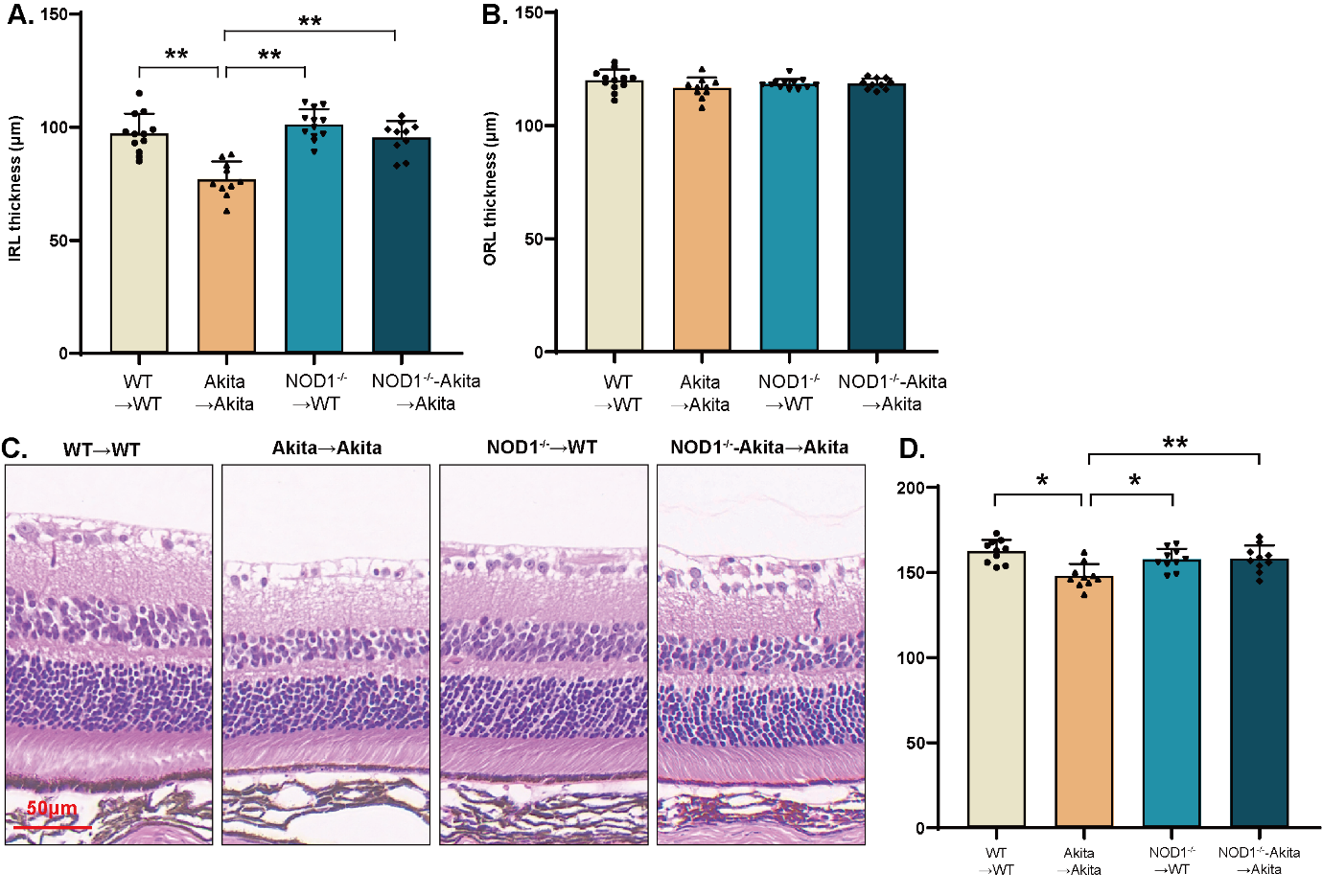


**Supplementary Figure 2: Retinal Thickness Changes in Bone Marrow Transplantation Mice in HE Staining.** (A-B) Quantification of IRL and ORL thickness by OCT. (C) Representative retinal HE staining in four different groups. (D) Retinal thicknesses by HE staining in different bone marrow transplantation mouse groups (n=10-12 per group). Results are presented as mean ± SD; *, p<0.05, **, p<0.01. Abbreviations: HE, hematoxylin-eosin staining; IRL, inner retinal layer; ORL, outer retinal layer.


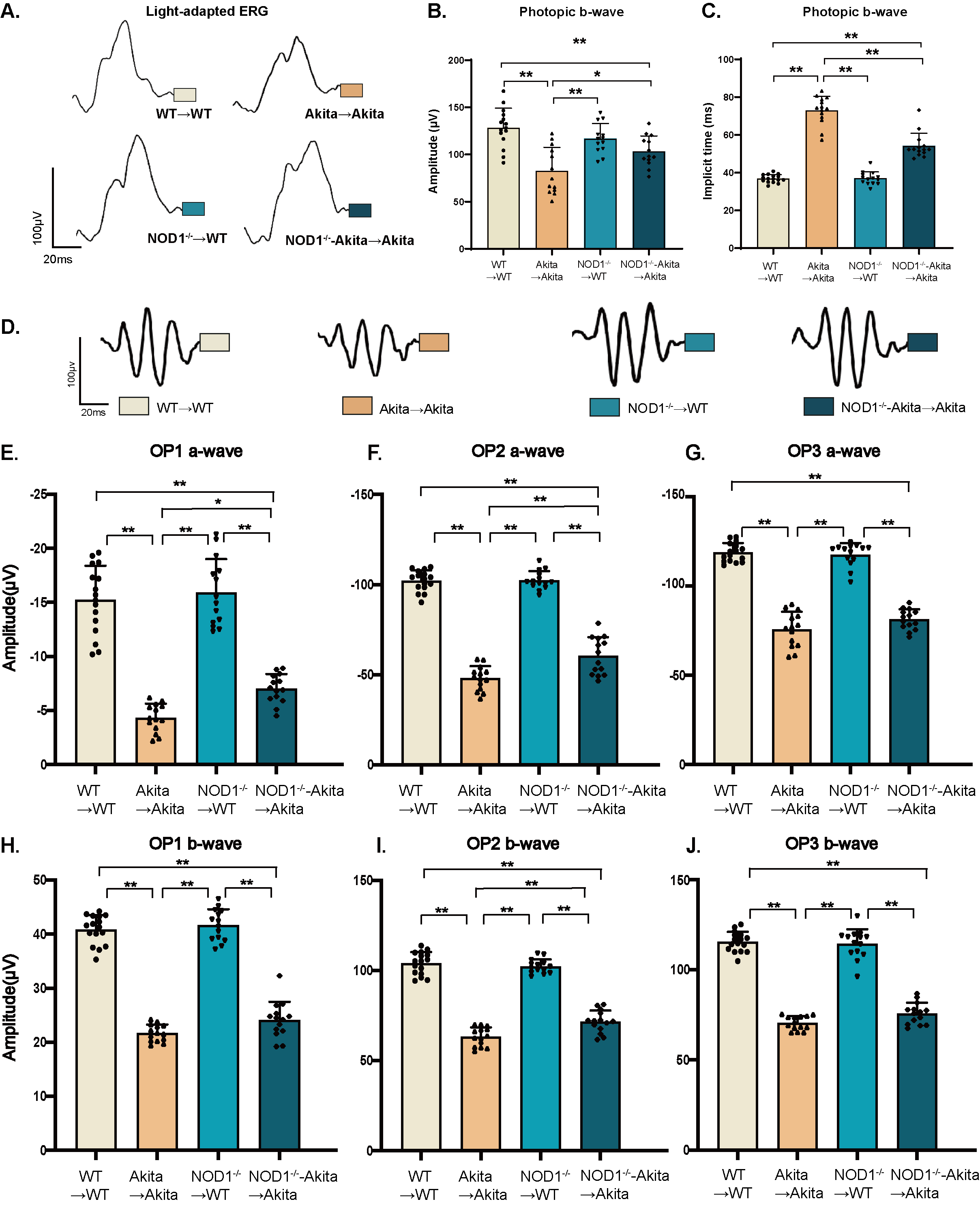


**Supplementary Figure 3: Hematopoietic-Specific NOD1 Knockout Alleviates Retinal Function in Photopic Conditions and Improves the Amplitudes of OPs in the Group with Diabetes.** (A) Representative photopic ERG waveforms generated by four groups with a flash intensity of 3.0 cd·s/m2 after light adaptation for 10 minutes. (B) Comparison of the b-wave amplitudes in photopic conditions respectively (n=14-16 per group). (C) The improvement in retinal function in NOD1-/--Akita→Akita was associated with a decrease in implicit times of the b-wave (n=14-16 per group). (D) Representative OPs waveforms from each bone marrow transplantation group. (E-J) The differences of OPs a-wave and b-wave in different groups (n=14-16 per group) were analyzed. Results are depicted as mean ± SD; *, p<0.05, **, p<0.01. Abbreviations: ERG, electroretinogram; OPs, oscillatory potentials.
